# Supplementary material for: Identification of Immune Function-Related Subtypes in Cutaneous Melanoma
Source: Life (Basel). 2021 Sep 6;11(9):925. doi: 10.3390/life11090925 (PMC8467264; doi:10.3390/life11090925)

Supplementary Figures

Supplementary Figure S1: Heatmap of tumor microenvironment in four subtypes: normal, immune1, immune2 and immune3.

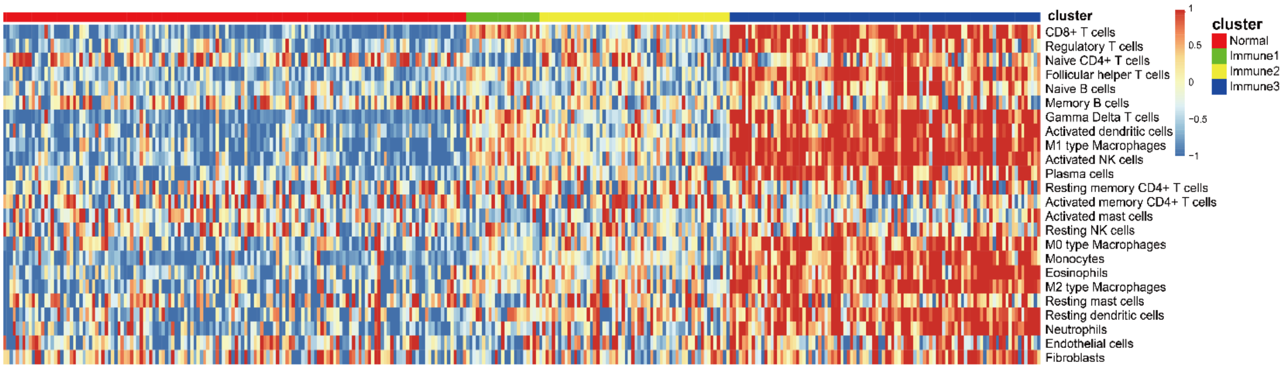

Supplementary Figure S2: There was a significant different level of immune score among four subtypes ($p < 0.05$).

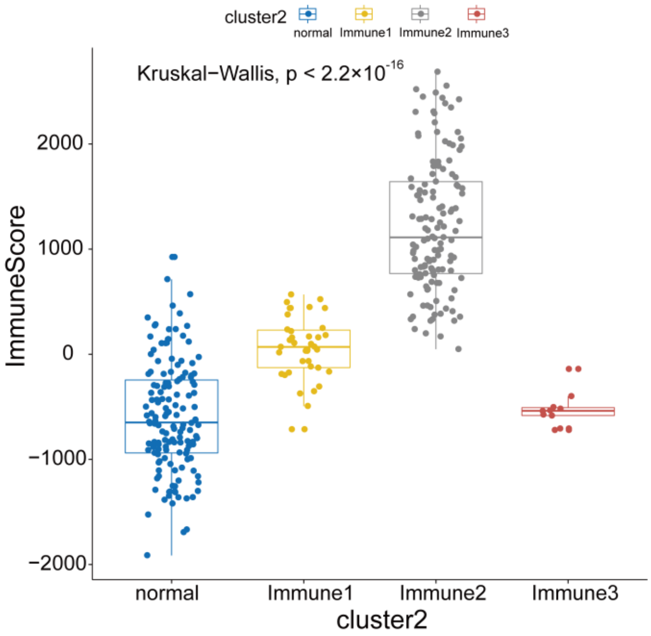

Supplementary Figure S3: Th1, Th2 and Th17 cytokines abundances and expression of PD-L1 genes showed an increasing trend among “immune inactivation subtype”, “low immune subtype” and “high immune subtype”. The abundance of MDSC was the highest in “immune inactivation subtypes” and lowest in “high immune subtype”.

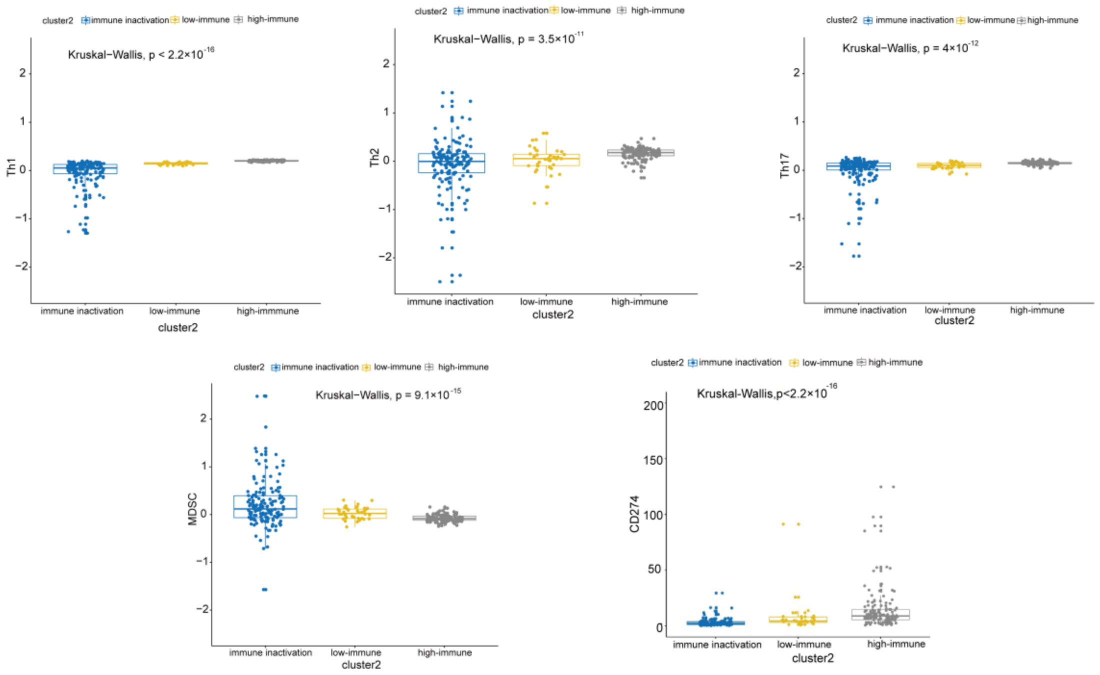

Supplementary Figure S4: Waterfall map of significantly mutated genes in “Normal class” (n=145), “Immune class” (n=180), “low-immune subtype” (n=38) and “high-immune subtype” (n=132).

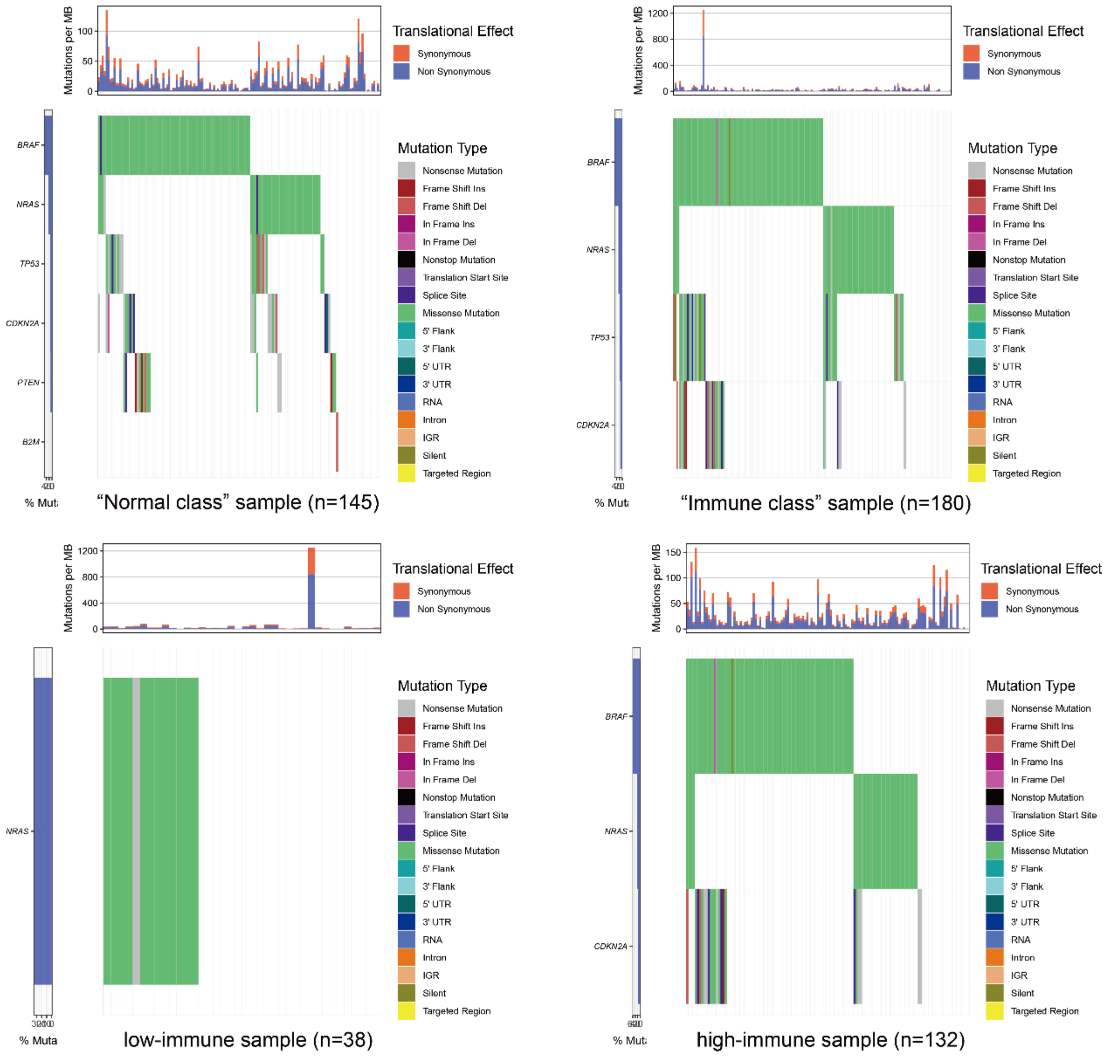

Supplementary Figure S5: Significantly differences of TMB levels were manifested normal and immune classes ($p < 0.05$).

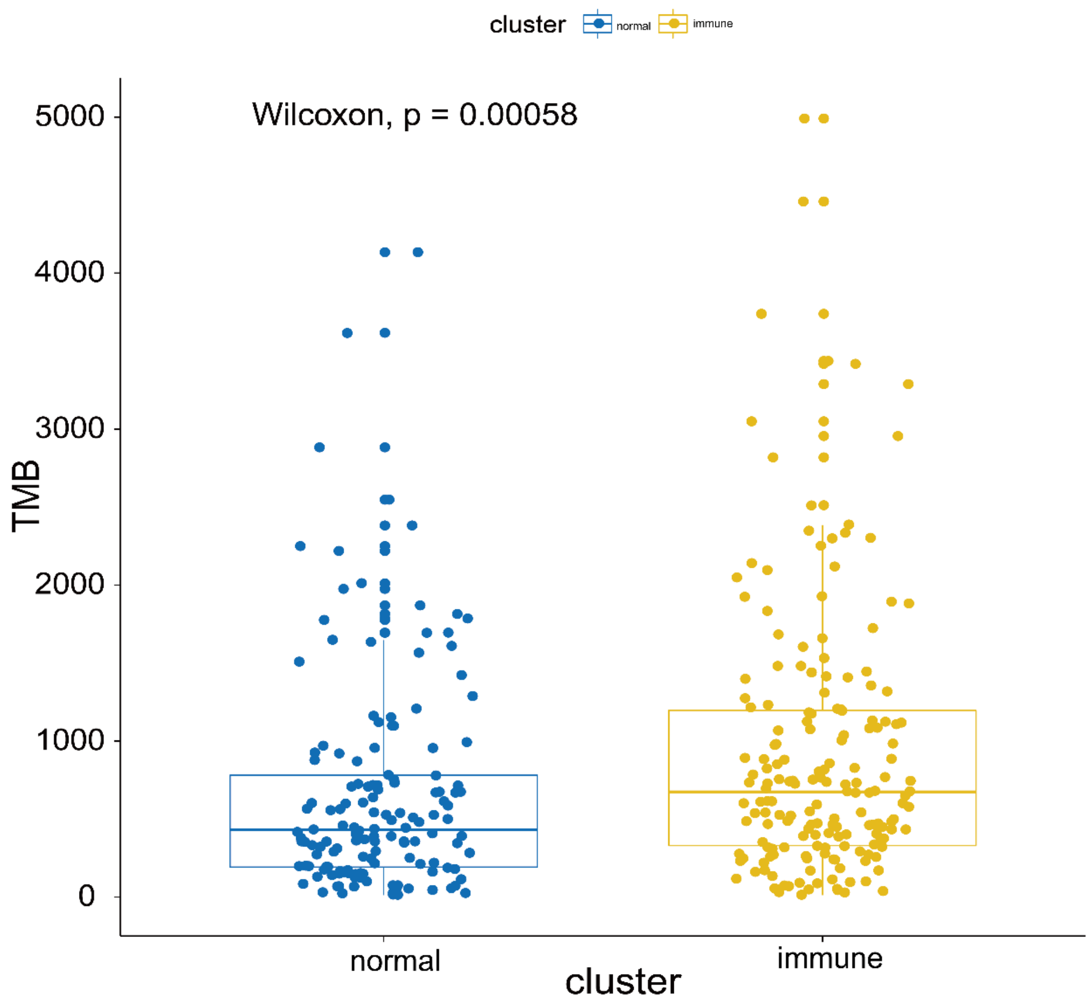

Supplementary Figure S6: The normal and immune classes identified by the validation set. There were significantly differences between normal and immune classes in cell cytotoxicity and cytolysis, immune score and stromal score.

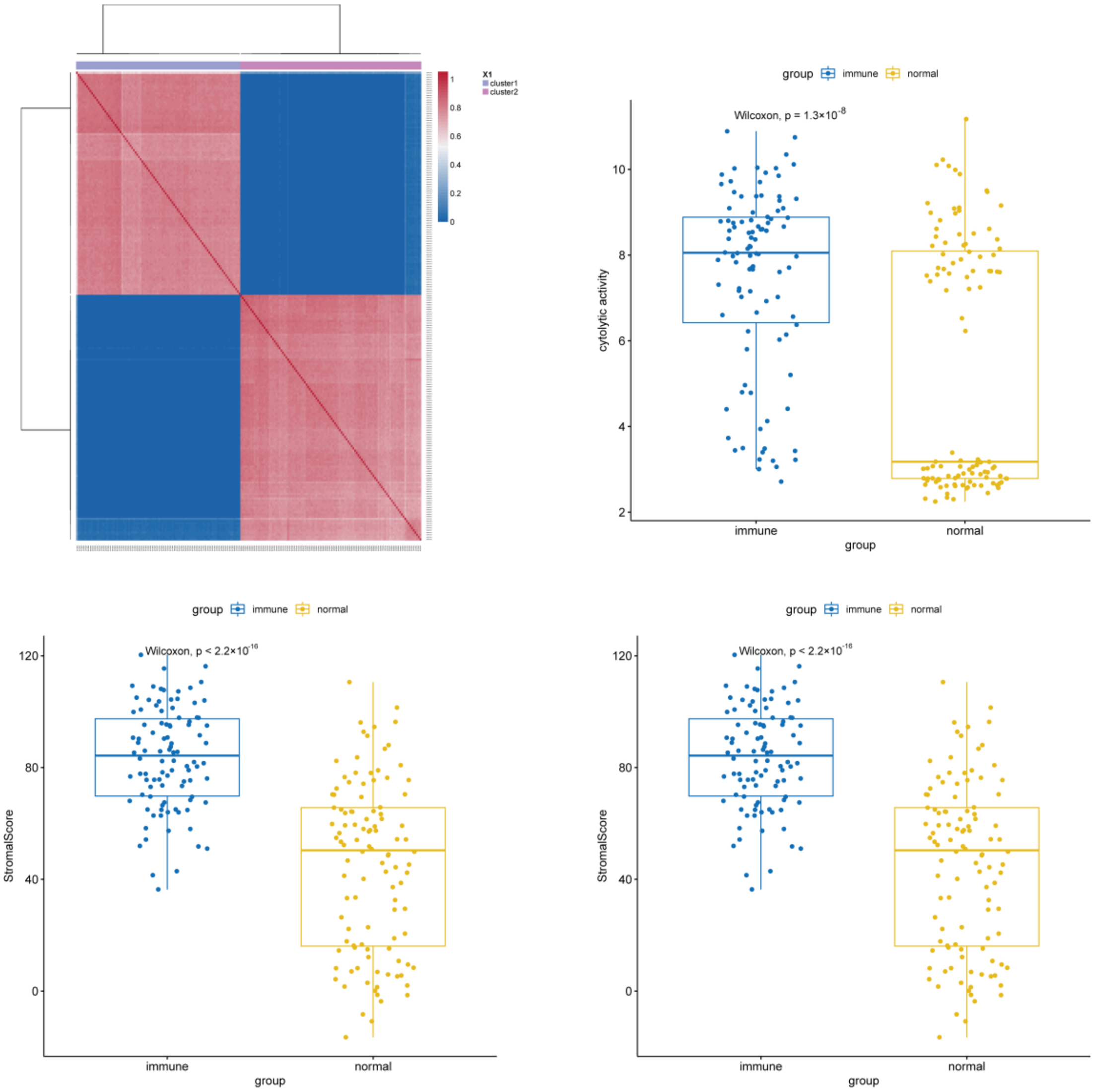

Supplementary Figure S7: Determination of the optimal cluster number (k = 3) according to BIC values in the cell lines data. There was no significant differences among the three clusters in the cell cytotoxicity and cytolysis (P = 0.51) because the gene expression of the **GZMA**, **PRF1** were too low.

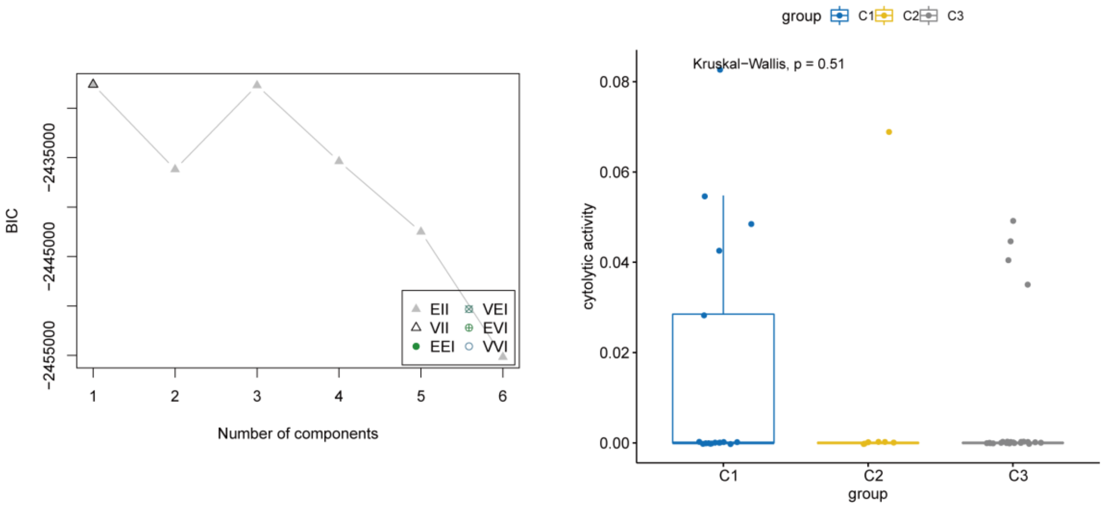

Supplementary Figure S8: There were significant differences among the three immune subtypes in the riskscore (P < 0.05) according to different conditions. “High immune subtype” had a lower risk score than other two subtypes.

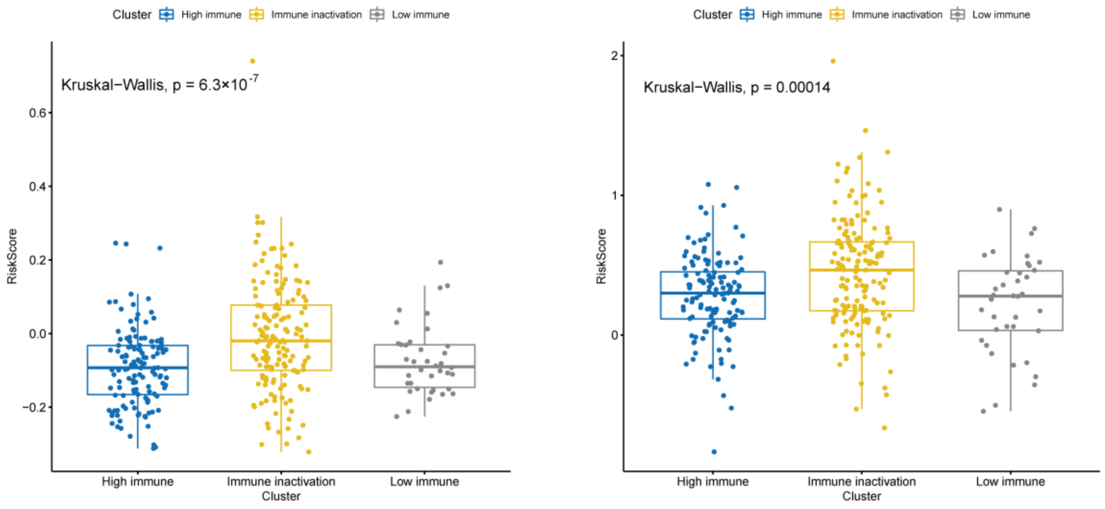

Supplement: Supplementary file 1 [file life-11-00925-s001.zip › revision_Supplementary files/life-1355810-Manuscript.Supplementary Figures.pdf]
